# Unravelling the Mystery of Psoriasis Dermatitis (PsoDermatitis): A Practical Guide to Recognition and Management

**DOI:** 10.3390/jcm15010130

**Published:** 2025-12-24

**Authors:** Riccardo Balestri, Michela Magnano, Salvatore Domenico Infusino, Tommaso Ioris, Rossella Lacava, Carlo Rene Girardelli, Giulia Rech

**Affiliations:** 1 Division of Dermatology, Psoriasis Outpatient Service, Azienda Sanitaria Integrata del Trentino (ASUIT)—Trento Hospital, 38122 Trento, Italy; riccardo.balestri@apss.tn.it (R.B.); salvatore.infusino@gmail.com (S.D.I.); tommaso.ioris@apss.tn.it (T.I.); rossella.lacava@apss.tn.it (R.L.); carlorene.girardelli@apss.tn.it (C.R.G.);; 2 Unit of Dermatology, Lucca Hospital, Azienda USL Toscana Nord Ovest, Via Guglielmo Lippi Francesconi 556, 55100 Lucca, Italy

**Keywords:** psoriasis, atopic eczema, biologics

## Abstract

Historically, psoriasis (Pso) and atopic dermatitis (AD) were considered mutually exclusive diseases due to their activation of opposing immune pathways—Th17 in Pso and Th2 in AD. However, growing clinical evidence and systematic reviews have challenged this dichotomy, demonstrating that both conditions can coexist in the same individual. This paper aims to provide an overview of the nomenclature, epidemiology, genetics, pathogenesis, and clinical features of coexistent psoriasis and atopic dermatitis, whether drug-induced or not. These conditions are also collectively referred to as “PsoDermatitis”.

## 1. Introduction

Historically, psoriasis (Pso) and atopic dermatitis (AD) were regarded as mutually exclusive diseases due to their activation of opposing immune pathways: Th17 and Th2, respectively. However, increasing clinical evidence and systematic reviews have challenged this dichotomy, demonstrating that both conditions can coexist in the same individual.

We propose a guide for understanding, recognizing, and managing this broad group of paradoxical inflammatory skin manifestations.

## 2. Materials and Methods

An extensive search of the literature was conducted using the PubMed, Scopus, Embase, and MEDLINE databases, and included studies published up to August 2025. The search approach integrated MeSH terms with free-text keywords related to “psoriasiform dermatitis”.

In particular, the strategy encompassed terms addressing the disease’s underlying mechanisms, clinical manifestations, diagnostic standards, histopathological characteristics, and therapeutic approaches. Additional keywords referring to specific molecules and therapeutic strategies were included, such as “dupilumab”, “JAK inhibitors”, “biologics”, and “biological drugs”.

Only articles published in English that met criteria for clinical relevance and methodological rigor were included, encompassing reviews, meta-analyses, preclinical studies, case reports, and real-world data. The reference lists of the included papers were also examined to identify other relevant publications. This review is based solely on previously published literature and did not involve any original research involving human or animal subjects.

Two authors (R.B. and M.M.) independently screened titles and abstracts, followed by full-text assessments. Any discrepancies were resolved through discussion and consensus.

## 3. Results

### 3.1. Nomenclature

While the first studies investigating concomitance of Pso and AD can be traced to the early 1990s [1], the very first article that tried to define an intermediate phenotype featuring lesions with characteristics of both psoriasis and eczema was published in 2005, and the authors suggested naming this category of inflammatory dermatosis “PsEma” [2].

Subsequently, this name was brought back in 2019 with the first report of PsA and AD treated with a biological combination [3]; however, the articles that followed proposed a number of synonyms, including “Psoriasis Dermatitis”, “Psoriasis and Atopic dermatitis concomitant disease”, “Psoriasis-Atopic Dermatitis overlapping syndrome”, “Eczema in psoriatico”, “PSO-Eczema”, and “Psorema” [4].

The terms eczema and AD are often used interchangeably, but they are not exactly equivalent. Eczema is a broad, non-specific term used to describe a group of inflammatory skin conditions that share similar symptoms, while AD is a specific type of eczema, which is chronic, relapsing, and is associated with a genetic predisposition.

Therefore, all the terms refer directly to eczema should be avoided when referring to to the condition described here.

Consequentially, although this condition has been described by various names, we have chosen to use the term PsoDermatitis throughout this text, since it represents a simple, immediate, and easily recognizable contraction of the more descriptive name Psoriasis Dermatitis.

### 3.2. Epidemiology

#### 3.2.1. Non-Drug-Related PsoDermatitis

The incidence and prevalence of PsoDermatitis remain extremely difficult to accurately define.

Due to the different nomenclature used throughout the years and the relatively recent recognition of the spectrum of PsoDermatitis, at present, there are no large cohort studies that have assessed the real prevalence of this disease in the Caucasian population.

Moreover, heterogeneity was high, potentially due to differences in geographical location and diagnostic method. For example, higher prevalence was observed in Europe and in studies with dermatologist-confirmed diagnoses: when diagnosing physicians were blinded to the study hypothesis, lower prevalence values were reported in contrast to much higher rates in non-blinded studies [4].

Since most cases of PsoDermatitis are drug-related (such as anti-TNF-α, anti-IL-4/12/13/17/23), as will be explained later, the introduction of biologics for the treatment of Pso (Etanercept, which was FDA-approved in April 2004) and AD (Dupilumab, which was FDA-approved in March 2017) has undoubtedly contributed to the substantial increase in the number of reported cases.

In fact, while in the past, patients with Pso were reported to have a 25-fold lower prevalence of AD [5], a recent Taiwanese study has shown a bidirectional association between PSO and AD [6].

A systematic review by Cunliffe et al. [4] found that the prevalence of AD in patients with Pso ranged from 0.17% to 20%, with a pooled estimate of 2%. Similarly, the prevalence of Pso in patients with AD ranged from 0.3% to 12.6%, also yielding a pooled estimate of 2%. These findings were echoed by Barry et al. [1], who reported a 1.3% prevalence of concomitant AD and Pso in a mixed population.

#### 3.2.2. PsoDermatitis as a Paradoxical Reaction (Drug-Related PsoDermatitis)

PsoDermatitis could represent a paradoxical inflammatory condition that manifests during targeted biologic treatments.

- PsoDermatitis associated with biological drugs used to treat Pso

The possibility of developing AD as a side effect of anti-TNF-alpha therapy became immediately evident from the first use of infliximab in the fields of rheumatology [7] and dermatology [8].

According to a review focused on this class of biologics, AD and eczema occurred as adverse effects in approximately 5–20% of patients with various Th1-mediated inflammatory conditions (e.g., psoriasis, arthritis, and inflammatory bowel disease), with infliximab showing the strongest association with the development or exacerbation of pre-existing AD [9].

Similarly, the Danish biologics registry (DANBIO) found that 26.1% of rheumatology patients (n = 419) who started taking infliximab or etanercept developed eczema [10].

A subsequent review, which also included anti-IL-12/23 agents as well as secukinumab and ixekizumab, reported this adverse event in 1.0–12.1% of patients based on trial data and observational studies, with a higher prevalence observed for anti-IL-17 therapies [10].

Finally, a recent analysis of data from the British Association of Dermatologists reported paradoxical PsoDermatitis related to biologic therapy in approximately 1% of psoriasis patients, with incidence rates varying across drug classes. The highest incidence was observed in patients receiving IL-17 inhibitors, followed by those on TNF inhibitors, IL-12/23 inhibitors, and IL-23 inhibitors. Compared to TNF-alpha inhibitors, exposure to IL-23 inhibitors was associated with a significantly lower risk of paradoxical eczema, whereas no significant difference in incidence was observed between TNF-alpha inhibitors and IL-17 inhibitors [11].

Older age, female sex, and hay fever were identified as risk factors for the development of PsoDermatitis, even though a personal history of atopic diseases (in particular AD) represented the strongest risk factor, identified by almost the studies [11].

- PsoDermatitis associated with biological drugs used to treat AD

Inhibition of IL-4 and IL-13 has been associated with new-onset or exacerbation of Pso.

Incidence rates of dupilumab-associated PsoDermatitis vary across studies. The largest cohort, in a study by Jaulent et al. [12], reported an incidence of 1.88% (7 out of 373 patients). Napolitano et al. reported marginally higher frequencies, with rates of 3.33% (3/90) in 2019 [13] and 3.03% (5/165) in 2021 [14]. Although these percentages are comparable to the prevalence of classical psoriasis in the general population (ranging from 0.36% to 2.73% according to geographic and demographic variables) [15], this finding might indicate a coincidental relationship. However, a greater incidence was documented in a retrospective analysis, in which psoriasis occurred in 7.0% of patients treated with dupilumab (14 out of 199 cases) [16].

Notably, 43% (6 out of 14) of these patients had a prior history of psoriasis, potentially explaining the elevated rate and reinforcing the recommendation to avoid dupilumab in individuals with a known history of Pso.

Intriguingly, these values are closely aligned with the reported incidence of non-drug-related PsoDermatitis, suggesting that intrinsic predisposition may play a key role, while dupilumab might serve as an extrinsic trigger in susceptible individuals.

A scoping review found 44 patients that developed de novo psoriasis, while 3 experienced flares of pre-existing psoriasis after initiation of dupilumab treatment, with an approximately equal distribution of males and females.

The mean time to onset of de novo psoriasis in adults was 4.3 months (range: 1–18 months), which is in line with findings from earlier reports, while for those with flare-up of pre-existing psoriasis, the average onset occurred sooner, at about 3.3 months.

Notably, most cases occurred within the 1st year of dupilumab therapy, even though isolated cases with delayed onset have been observed 1.5 and even 2.5 years after treatment initiation [17].

### 3.3. Genetics

Numerous genetic loci have been identified for each condition, reflecting their genetic heterogeneity. The primary susceptibility gene for psoriasis is HLA-Cw*0602 located on the PSORS1 locus (6p21), while for AD, one of the most significant genetic risks is associated with null mutations in the filaggrin gene (FLG) [18].

Genetics may play a significant role as a risk factor for the development of both disorders. More than 80% of dysregulated genes found in lesional skin of patients with AD have also been identified in Pso [19]. Linkage studies have demonstrated shared susceptibility loci between AD and Pso [20]. The risk of developing both conditions appears to be higher when first-degree relatives are affected by either AD or Pso [6].

Conversely, genome-wide association studies support the idea that Pso and AD are generally mutually exclusive, as common risk alleles for psoriasis tend to exhibit opposing risk profiles for AD.

However, one study identified several alleles that confer similar risk levels for both Pso and AD. Given that individual genetic variants can have pleiotropic effects, some researchers have speculated that carriers of these specific alleles may be more susceptible to developing AD concurrently, particularly under certain conditions such as exposure to biologic therapies [10]. Moreover, comparative genomic studies have revealed conflicting findings in overlapping chromosomal regions. The epidermal differentiation complex (EDC) on chromosome 1q21 [3], which houses FLG mutations relevant to AD, shows no relation to PSO. Conversely, LCE3B/3C gene deletions, which are also located in the EDC and linked to PSO, are not associated with AD. Interestingly, some studies reported that certain FLG variants may increase the risk of psoriasis in Taiwanese and Chinese populations, suggesting possible ethnic differences in genetic susceptibility [18].

### 3.4. The Immunological Spectrum

Psoriasis is a disease primarily driven by Th17 T-cells and associated IL-17 activation, whereas atopic dermatitis (AD) is characterized by a strong Th2 response with overproduction of IL-4 and IL-13. Both diseases also involve activation of Th22 cells and Th1 pathways, leading to increased production of IL-22 and IFN-γ, respectively.

In particular, although AD has traditionally been considered a Th2-driven inflammatory disorder, current evidence reveals that AD involves the activation of multiple T-cell subsets at different disease stages. The acute phase is marked by prominent Th2/Th22 activation, while the chronic phase sees a progressive increase in Th1/Th17 activity, resembling the immunological profile of psoriasis. Importantly, this transition does not represent a replacement of Th2/Th22 by Th1/Th17, but rather their coactivation and persistence over time. Although IL-17-producing cells are more abundant in psoriasis, their levels in severe AD are comparable, suggesting a shared effector mechanism that may underlie overlapping clinical and pathological features between chronic AD and psoriasis [19,20,21].

Recent studies suggest that the Th17–Th2 dichotomy may actually represent a continuum, allowing for dynamic immune shifts between the two ends [22].

In Caucasian patients, overlapping clinical features of AD and Pso have been observed, suggesting that these conditions may represent a spectrum of disease despite their distinct immune polarizations.

Furthermore, co-occurrence of both conditions has been reported, and phenotypic switching between AD and Pso can happen either spontaneously or during biologic therapy—a phenomenon recently defined as the “flip-flop” phenomenon [23].

It has also been suggested that some of these cases are immunologically distinct from classic AD. Stoffel et al. identified that eczema and Pso arising in patients with inflammatory bowel disease and rheumatoid arthritis undergoing TNF inhibitor therapy exhibit unique immunological profiles compared to conventional forms of atopic eczema and psoriasis [24].

Pso and AD could potentially have an immunological intersection: similarly to Pso, the Th1, Th17, and Th22 pathways, as well as IL-36 receptor signaling, are frequently activated in AD, resulting in elevated cutaneous levels of IL-22, IFN-γ, IL-36, and IL-17.

IL-17C and IL-17E play significant roles in the pathogenesis of AD and in Th2-mediated inflammation, while IL-17A is a key driver of inflammation in Pso [23].

### 3.5. Pathogenesis

The hypothesis concerning the pathogenesis of AD and Pso revolves around the imbalance between Th1/Th17 and Th2 immune responses.

Several mechanisms may contribute to this switch, particularly immune pathway imbalance and cutaneous barrier defects that may favor overlap or transition between the two diseases, regardless the use of biologic therapies.

#### 3.5.1. Non-Drug-Related PsoDermatitis

Psoriasis and atopic dermatitis are both inflammatory dermatologic conditions driven by immune mechanisms involving T lymphocytes, cytokines, chemokines, and other immune cells. Within the skin, T-cells undergo differentiation into distinct subsets under the influence of specific cytokines and subsequently release mediators that modulate keratinocyte proliferation and differentiation, ultimately resulting in the development of diverse inflammatory skin disorders [25].

Eyerich et al. identified that antigen-specific T-cells migrate into the skin in response to allergen provocation and suggested that antigen triggers are responsible for determining the type of T-cell response and, therefore, the disease phenotype [26]. Similarly, Ono et al. identified that Pso autoantigens, cathelicidin leucine, leucine-37 (LL-37), and a disintegrin-like and metalloprotease domain containing thrombospondin type 1 motif–like 5 (ADAMTSL5), were more prominently expressed in plaques of psoriasis when compared with AD [27].

More recently, research has focused on the skin barrier–inflammatory pathway as a driver of the Pso-AD transition, regardless of the biologic therapies utilized.

Central to this interplay is the disruption of the skin barrier—via keratins, cornified envelope proteins, and tight junctions—which can activate reciprocal inflammatory loops [21]. Alterations in keratins such as K17 in psoriasis and K6 in AD, or in proteins like filaggrin and loricrin, can dysregulate local immune responses, fostering Th2 or Th17 polarization depending on the context [21]. Cytokine-driven feedback, especially involving IL-4, IL-13, IL-17, and IL-22, further modulates this loop, with IL-17A and IL-23 implicated in Th17-mediated inflammation and Th2 cytokines enhancing barrier dysfunction [21]. Biologic therapies may exacerbate this imbalance by selectively inhibiting one immune axis, thereby shifting the polarization—for example, IL-17A blockade favoring Th2 responses and potentially triggering AD, while Th2 blockade with dupilumab may promote Th17-driven psoriatic manifestations [21]. The emerging concept of a Th2–Th17 immunologic spectrum, rather than a dichotomy, is reinforced by findings of shared genetic susceptibility, overlapping AMP profiles (e.g., LL-37, S100A7), and the involvement of tissue-resident memory T-cells in relapse and chronicity [21]. Also, similarly to telangiectatic rosacea, the JAK/STAT signaling pathway, activated by LL-37, is crucial in both Pso and AD. Thus, psoriasis and AD may represent immunological poles within a common disease spectrum, where skin barrier integrity and immune polarization determine phenotypic expression and therapeutic response.

#### 3.5.2. Drug-Related PsoDermatitis

Pichler’s classification of biologics-induced adverse events includes type c events, described as immune or cytokine imbalance syndromes, which include atopic disorders and could explain the occurrence of AD in response to biologics [28].

- PsoDermatitis associated with biological drugs used to treat Pso

Since Pso is primarily driven by Th17 cytokines, with some involvement of Th1, and AD is Th2-driven, the most straightforward hypothesis is that blocking Th1/Th17 pathways may shift the immune balance toward Th2 dominance, potentially triggering AD-like phenotypes.

Since IL-17 inhibition is most strongly associated with the induction of PsoDermatitis, research has primarily focused on this class of drug, and three main hypotheses have been proposed.

Selective inhibition of IL-17A: selective inhibition of IL-17A, as seen with secukinumab and ixekizumab, may disrupt immune balance by permitting overexpression of other IL-17 isoforms, such as IL-17C, that are produced by epithelial cells and implicated in the pathogenesis of both Pso and AD [29,30].

Suppression of keratinocyte-derived antimicrobial peptides: IL-17C’s autocrine stimulation of keratinocytes is linked to both Th17- and Th2-driven inflammation, supporting its role in AD pathogenesis and the potential efficacy of IL-17C neutralizing antibodies. Munera-Campos M et al. suggested that the IL-17A inhibition may suppress the expression of keratinocyte-derived antimicrobial peptides, which could have a role at least in AD-like eruptions [31].

Interleukin-22 as a key mediator: Since 2011, it has been shown that IL-22 is produced by both Th17 and Th22 cells and is released in similar amounts in Pso lesions and AD lesions [26]. Data has shown high levels of IL-22 in PsoDermatitis induced by anti-TNF-alpha, compared with “pure” Pso and AD and a persistence of increased serum levels of IL-22 despite the use of IL-17A inhibitors. Therefore, IL-22 has been proposed as a potential trigger of PsoDermatitis [32].

- PsoDermatitis associated with biological drugs used to treat AD

PsoDermatitis caused by dupilumab displays mixed Th2-Th17 cytokine expression, indicating an intermediate inflammatory state.

It is well known that IL-4 is a cytokine that is capable of negatively affecting Th17 lymphocyte function and upstream IL-23 production secreted by antigen-presenting cells; therefore, IL-4/IL-13 inhibition may relieve suppression of the IL-23/IL-17 axis, increasing Th17-mediated inflammation and associated comorbidities like psoriasis, though not all Th17 diseases show this pattern, since ankylosing spondylitis and inflammatory bowel disease are not associated with dupilumab therapy [15].

Certain AD subtypes (e.g., Asian, pediatric) with intrinsic Th17 components may be more prone to developing psoriasis-like lesions during Th2 blockade [15].

A recent review analyzed 18 reported cases of dupilumab-induced psoriasiform dermatitis and showed that the majority of patients (83%) had a history of atopic dermatitis since childhood, indicating that long-standing AD may cause a predisposition to the development of psoriasis as a result of a partial shift toward Th1/Th17 immune pathways in chronic disease [33].

Interestingly, some studies have reported higher IL-23A expression and IL-17A production in dupilumab-induced PsoDermatitis, indicating activation of the IL-23/Th17 axis in psoriasiform lesions. Notably, the IL-17A levels were even higher than those observed in classic psoriasis, suggesting a pronounced Th17 shift triggered by dupilumab [15].

However, considering the relatively low frequency of PsoDermatitis, which is comparable to the prevalence of classic Pso and concomitant AD and Pso, it is more likely that dupilumab acts as a trigger rather than a causative agent in psoriasiform eruptions, with intrinsic susceptibility being the primary driver of disease expression.

### 3.6. Clinical and Histological Manifestations

**Figure 1:**
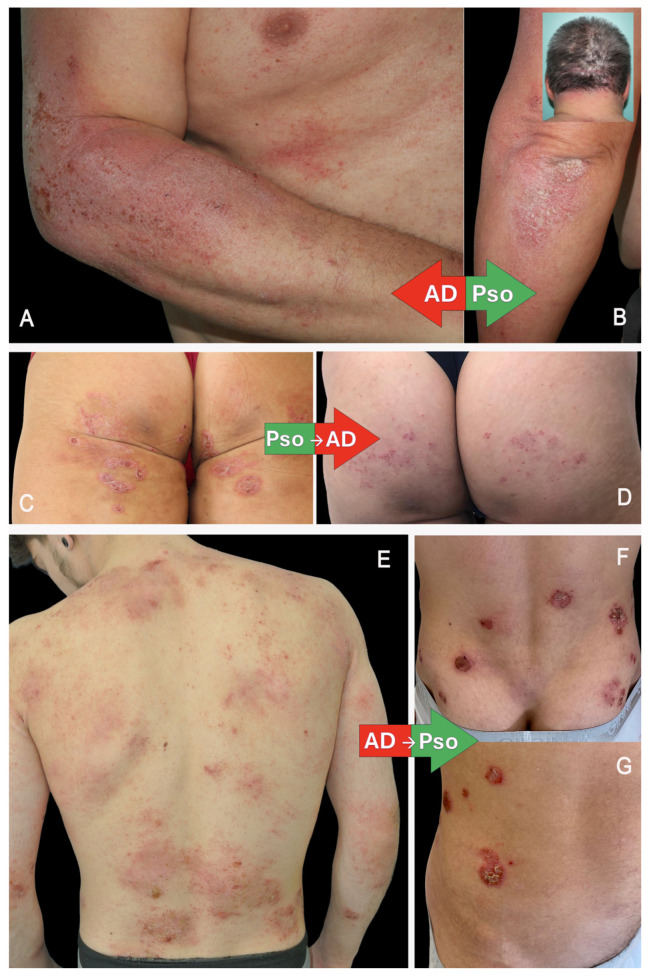
(**A**,**B**) Non-drug-related PsoDermatitis: coexistence of psoriasis and atopic eczema in the same patient; (**C**,**D**) drug-related PsoDermatitis, resulting in the development of AD after treatment with an anti-TNF-alpha; (**E**–**G**) drug-related PsoDermatitis, resulting in the development of Pso after treatment with dupilumab.

The clinical presentation represents an overlap between psoriasis and atopic dermatitis (Figure 1).

There are no specific hallmarks of the condition. An inversion of typical distribution patterns is often observed: psoriatic lesions may appear in flexural areas, while eczematous changes can be found on extensor surfaces. Clinical examination reveals mixed features characteristic of both diseases, and dermoscopic evaluation shows a variegated pattern of vascular structures and scaling.

The histopathological features of PsoDermatitis show a psoriasiform/spongiotic overlap. Key histopathologic features include irregular acanthosis, parakeratosis, mixed perivascular inflammatory infiltrates, and occasionally eosinophils [34]. In particular, the occasional presence of eosinophils and plasma cells has been reported in paradoxical psoriasiform plaques induced by anti-TNF-α therapy—a potential clue to distinguish them from classic psoriasis, which may also be useful in the diagnosis of PsoDermatitis [35].

#### 3.6.1. PsoDermatitis Associated with Biological Drugs Used to Treat Pso

Three main clinical/histopathological phenotypes have been described [36].

Classic acute eczema: Classical eczematous eruption during treatment with erythematous crusted and excoriated plaques on the trunk. Histopathology shows full-thickness epidermal spongiosis with lymphocytic exocytosis, spongiotic vesicles, and intracorneal serum crusts.

Psoriasiform eczema: Psoriasiform eczema-like eruption with erythematous hyperkeratotic excoriated plaques. Histopathology shows psoriasiform epidermal hyperplasia with mild spongiosis, parakeratosis, loss of the granular layer, and Munro intracorneal microabscesses.

AD-like eruptions: Atopic dermatitis-like with erythematous flexural plaques. Histopathology shows irregular acanthosis with minimal spongiosis and eosinophils in the dermis.

#### 3.6.2. PsoDermatitis Associated with Biological Drugs Used to Treat AD

PsoDermatitis induced by dupilumab resembles classic psoriasis in many aspects. Plaque psoriasis is the most commonly observed subtype; however, guttate, erythrodermic, pustular, inverse psoriasis, and sebopsoriasis have also been reported.

Interestingly, psoriatic lesions mainly appeared in cutaneous areas that had not been previously affected by atopic dermatitis. Nonetheless, some cases showed a direct transition from AD to psoriasis, with new psoriatic lesions developing on pre-existing eczematous sites. Scalp involvement was particularly common.

It is important to note that dupilumab can also trigger flare-ups of pre-existing psoriasis, often leading to more severe lesions and a shorter time to onset [15].

### 3.7. Clinical Implications and Management

The objectives in the management of PsoDermatitis are (i) recognition; (ii) prevention and (iii) treatment.

#### 3.7.1. Recognition: How to Perform a Differential Diagnosis

Recognizing this overlap is crucial for personalized care. While Muller et al. developed a clinical algorithm to predict the “Flip-Flop” phenomenon, with an extremely high specificity, they have not yet published this tool [23].

However, they described the ranking of the features of their initial model that performed well anyway, considering that it was not optimized with respect to the available data.

Table 1, adapted from the aforementioned article, lists the clinical aspects to consider when assessing PsoDermatitis.

Personal and family history should always be evaluated to identify any underlying diathesis.

#### 3.7.2. Prevention

- Before Receiving Biologics for Pso

It is well known that anti TNF-alpha and anti-IL-17 should be used carefully in patients with a history of atopy. A review found also that the development of eosinophilia or elevated IgE was reported in 46% and 38% of the included studies [10].

Consequentially, all the candidates for biologics for Pso should be investigated for prior history of atopy and periodically evaluated for the development of hematic markers of atopy.

- Before Receiving Biologics for AD

A history of psoriasis is strongly associated with the development of PsoDermatitis in AD patients undergoing dupilumab therapy. This finding highlights that dupilumab may not be suitable for patients with a prior history of psoriasis. In most cases, PsoDermatitis emerged within the first year of treatment, suggesting this period as a critical window for close clinical monitoring. However, isolated cases with delayed onset—occurring at 1.5 or even up to 2.5 years after dupilumab initiation—indicate that extended surveillance may be necessary in selected patients [17].

It is important to note that in children with a family history of Pso, early treatment of AD with biologic therapies may trigger the onset of PsoDermatitis. This observation raises intriguing possibilities regarding the potential to influence or modify the natural course of the disease through timely intervention.

### 3.8. Treatment (Figure 2)

#### 3.8.1. Non-Drug-Related PsoDermatitis

Conventional oral immunosuppressive therapy and phototherapy can be used to treat both AD and PSO, while biologic agents targeting only specific T-cells or cytokines are often ineffective for concurrent diseases and are more likely to induce PsoDermatitis.

Recent studies have highlighted the potential efficacy of JAK inhibitors (JAKis) in the management of PsoDermatitis [37], since this pharmacological class exerts broader immunomodulatory effects, inhibiting both Th1 and Th2 pathways.

**Figure 2:**
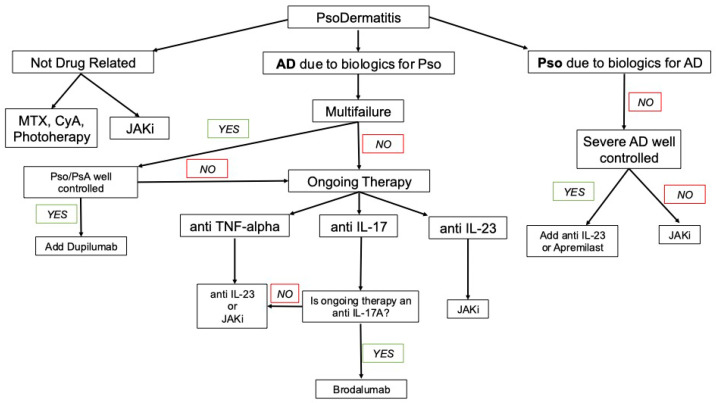
Flow chart for the suggested management of PsoDermatitis.

At present, there are no available JAKis that have been approved to treat both cutaneous Pso and AD.

We have found case series related to the treatment of PsoDermatitis with various JAKis; however, the only molecule with proven efficacy supported by clinical Phase III trials in both diseases is upadacitinib. In fact, while this JAKi is registered for the treatment of AD and psoriatic arthritis, it has been associated with a high proportion of patients achieving PASI75, PASI90, and PASI100 in randomized controlled trials and also showed superiority over adalimumab in achieving PASI75 [38,39].

Notably, although it is extremely rare, JAKis are not entirely devoid of this paradoxical effect, as one case of PsoDermatitis has been reported in response to treatment with upadacitinib [40].

#### 3.8.2. Drug-Related PsoDermatitis

JAKis represent a viable option not only for non-drug-related PsoDermatitis, but also for drug-related PsoDermatitis; however, when we are facing a paradoxical reaction, other strategies are available.

- Combination of Monoclonal Antibodies (CMAs)—combined targeted therapies that comprise both immunological axes. If the underlying disease for which biological therapy was initiated is well controlled—particularly in the case of multi-failure patients—the addition of another targeted therapy appears to be a reasonable option. This approach has been shown to be safe and effective, although it raises concerns with regard to economic sustainability [41].
- PsoDermatitis associated with anti TNF-alpha—consider JAKis, CMAs or a swap for anti-IL-23. The latter is based on the observation that paradoxical eczema risk seems to be lowest in patients receiving IL-23 inhibitors [11].
- PsoDermatitis associated with anti-IL-17—consider JAKi, CMA or a swap for anti-IL-23. In the case of PsoDermatitis associated with anti-IL-17A, broader IL-17 pathway inhibition may help to prevent this adverse event; therefore, the use of brodalumab may be considered, as it targets the IL-17 receptor A (IL-17RA) and thereby blocks multiple IL-17 isoforms, including IL-17C and IL-17E (the latter is involved in Th2-mediated inflammation).
- PsoDermatitis associated with anti-IL-23: consider JAKi or CMA.
- PsoDermatitis associated with anti-IL-4/13: Consider JAKi or CMA. Despite the paucity of reported cases—and due to the recent approval of these drugs—the development of PsoDermatitis has also been associated with selective IL-13 inhibition by tralokinumab. To date, no such reports have been documented for lebrikizumab [42].

## 4. Conclusions

The coexistence of AD and Pso challenges the traditional view of mutually exclusive inflammatory pathways.

The theory of T-cell polarization imbalance may represent a central mechanism in PsoDermatitis, with common genetic and environmental factors linking AD and psoriasis. Although the simultaneous elevation of IL-17/IL-23 and sustained IL-4/IL-13 expression appears paradoxical, it underscores that PsoDermatitis is a distinct inflammatory condition characterized by an intermediate Th2–Th17 state. This is also supported by observations in Asian patients with AD, in whom IL-17 levels were found to be significantly elevated. Moreover, intrinsic AD, which differs from extrinsic AD in having normal serum IgE levels, is characterized by a heightened capacity for IL-17 production [43].

PsoDermatitis is a compelling example of how modern targeted therapies can reshape disease phenotypes through unintended immune modulation. Clinicians should remain vigilant, especially during the first year of biologic treatment, and adopt a flexible therapeutic approach tailored to each patient’s evolving immunologic profile.

## Figures and Tables

**Table 1:** Ranking of psoriasis and atopic dermatitis clinical features.

Ranking of Psoriasis and Atopic Dermatitis Clinical Features		
	Psoriasis	Atopic Dermatitis
** 1 **	Erythrosquamous plaques on the body and/or extremities *	Eczema and/or lichenification of the flexors *
** 2 **	Pustules (facial pustules excluded) *	Dyshidrotic eczema *
** 3 **	Erythema of the rima ani	Dennie–Morgan fold and/or orbital darkening
** 4 **	Scalp infestation beyond the forehead hairline	Perlèche (angular cheilitis) and/or cheilitis *
** 5 **	Plaques psoriasis localized retroauricular	Head and neck dermatitis and/or dirty neck
** 6 **	Psoriatic nail changes (pitting, oil-drop spots, nail plate crumbling)	Keratosis pilaris
** 7 **	Dactylitis/enthesopathy	Personal history for atopy (AST, AD, ARC)
** 8 **	Exacerbation after discontinuation of systemic steroid therapy	Sensitizations/food allergies
** 9 **	Family history of psoriasis	Palmar hyper-linearity
** 10 **	Joint pain	Family history of atopy (AST, AD, ARC, “eczema”)

* = currently or in the past; AD = atopic dermatitis; AST = asthma, ARC = allergic rhinoconjunctivitis.

## Data Availability

Data are available upon reasonable request.

## Abbreviations

The following abbreviations are used in this manuscript:

AD	Atopic Dermatitis.
Pso	Psoriasis.
JAKs	Janus Kinases.
CMA	Combination of Monoclonal Antibodies
